# Chinese medicine in the treatment of autoimmune hepatitis: Progress and future opportunities

**DOI:** 10.1002/ame2.12201

**Published:** 2022-01-19

**Authors:** Jia Liu, Zhi Ma, Han Li, Xiaojiaoyang Li

**Affiliations:** ^1^ 47839 School of Life Sciences Beijing University of Chinese Medicine Beijing China

**Keywords:** animal models, autoimmune hepatitis, immune regulation, Traditional Chinese medicine

## Abstract

Autoimmune hepatitis (AIH) is a chronic inflammatory liver disease occurring in individuals of all ages with a higher incidence in females and characterized by hypergammaglobulinemia, elevated serum autoantibodies and histological features of interface hepatitis. AIH pathogenesis remains obscure and still needs in‐depth study, which is likely associated with genetic susceptibility and the loss of immune homeostasis. Steroids alone and in combination with other immunosuppressant agents are the primary choices of AIH treatment in the clinic, whereas, in some cases, severe adverse effects and disease relapse may occur. Chinese medicine used for the treatment of AIH has proven its merits over many years and is well tolerated. To better understand the pathogenesis of AIH and to evaluate the efficacy of novel therapies, several animal models have been generated to recapitulate the immune microenvironment of patients with AIH. In the current review, we summarize recent advances in the study of animal models for AIH and their application in pharmacological research of Chinese medicine‐based therapies and also discuss current limitations. This review aims to provide novel insights into the discovery of Chinese medicine‐originated therapies for AIH using cutting‐edge animal models.

## Graphical abstract

Generalized relations between traditional Chinese medicines and different animal models for AIH.

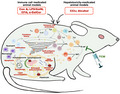

## 1 INTRODUCTION

Autoimmune hepatitis (AIH), a chronic self‐perpetuating inflammatory liver disease with a female preponderance, has been characterized by moderate or severe interface hepatitis, hypergammaglobulinemia, elevated serum aminotransferase and autoantibodies, hepatocyte necrosis and even liver failure owing to immunological tolerance.^1^ Comparing cases over past decades, the incidence of AIH has sharply increased in individuals of all ethnicities, regions and ages.^2^, ^3^ Due to the lack of specific and well‐accepted biomarkers, AIH diagnosis is based on diagnostic criteria comprising a combination of multiple parameters and is distinct among different age groups.^4^ Based on numerous clinical reports, AIH is more likely to be initiated and rapidly developed in female adults and is characterized by mild fatigue, elevated levels of γ‐globulins (mainly IgG), increased autoantibodies that react with intrahepatic and extrahepatic antigens or fulminant hepatitis, in some cases.^5^ Unlike in adults, AIH in children and adolescents shows a cascade of T‐cell‐medicated reactions that target hepatocytes, resulting in anorexia, headache, repetitive jaundice and progressive fatigue but not fulminant hepatitis.^6^ If continuously left untreated, AIH results in destruction of hepatic parenchyma and persistent hepatitis, and probably evolves into cirrhosis, hepatobiliary malignancies and even multiple organ failure in severe cases.^5^, ^7^, ^8^

At present, AIH is recognized as two types: AIH‐1, positive for anti‐nuclear (ANA) and/or anti‐smooth muscle (SMA) antibodies, and AIH‐2, positive for anti‐liver kidney microsomal antibody type 1 (anti‐LKM1), anti‐LKM3 and/or anti‐liver cytosol antibody type 1 (anti‐LC1).^9^, ^10^ Recent studies have revealed that the etiology and pathogenesis of AIH are correlated with genetic susceptibility, the loss of self‐tolerance, molecular mimicry or non‐specific activation of T lymphocytes.^11^ Notably, the interaction between genetic susceptibility and the loss of self‐tolerance caused by environmental factors is the core of pathogenesis.^12^ In adults, genetic predisposition to AIH is strongly associated with the specific genes located within the human‐leukocyte‐antigen (HLA) region on the short arm of chromosome 6, especially with genes encoding HLA‐DR4 and HLA‐DR3 alleles.^13^ In patients with HLA‐DRB1 cross‐reactions with liver autoantigens and rapid disease progression are more likely to occur.^14^ In addition, several environmental factors including drugs, xenobiotics and viruses result in the loss of self‐tolerance and initiate the immune response, and current studies aim to address this issue by developing animal models that mimic the liver damage caused by the immune system dysfunction in AIH patients.^15^ T lymphocytes control the balance of intrinsic and extrinsic peripheral tolerance in healthy individuals, alteration of which may give rise to the dysregulation of immunoregulatory networks. Support for the role of loss of self‐tolerance in AIH comes from studies showing that the deletion of medullary thymic epithelial cells that regulate T cell tolerance by eliminating autoreactive T cells and expressing self‐antigens caused the enrichment of anti‐ANA and initiated AIH in mice.^16^, ^17^ Moreover, antigen‐presenting cells (APCs) are involved in the process and presentation of self‐antigens to T cell receptor (TCR) on naive CD4^+^ T helper (T_H_0) cells and trigger immunoregulation. Exposure to self‐mimicking exogenous sequences results in the production of anti‐LKM1 antibodies in patients who have α1‐antitrypsin deficiency and acquired hepatitis C virus after liver transplantation.^18^, ^19^ Cytochrome P4502D6 (CYP2D6) is reported to mainly express in endoplasmic reticulum and hepatocyte membranes and become the target of anti‐LKM1 antibodies, thus leading hepatocytes to be directly attacked by humoral immunity.^20^

At present, first‐line therapeutic drugs targeting AIH and complications are glucocorticoids and azathioprine, which can be used either alone or in combination to achieve biochemical remission, defined as normal serum transaminase and IgG levels.^21^, ^22^ However, patients with AIH generally relapse after cessation of these drugs or suffer side effects caused by their long‐term use.^23^ When AIH progresses into end‐stage cirrhosis or fulminant liver failure, patients are no longer sensitive to pharmacotherapy and are forced to choose the final treatment option, liver transplantation.^24^ Therefore, exploring novel medications with high efficiency and low toxicity is of both basic science and clinical relevance. Traditional Chinese medicine (TCM) consists of different medicinal formulas and has shown success in regulating immune responses, thus exhibiting therapeutic effects against acute or chronic liver diseases.^25^ Considering the multi‐component and multi‐target characters of TCM, identifying various Chinese herbal prescriptions or natural Chinese medicine extracts is potentially an efficient approach to discovery of novel therapeutics against AIH. Recently, more AIH patients have prefered to take TCM to alleviate their severe symptoms and suppress steroid‐induced side effects.^26^ Many experimental studies have illustrated the promising therapeutic effects of several natural products from TCM, including phenols, glycosides, flavonoids, polysaccharides and alkaloids, in the treatment of AIH by activating T/B lymphocytes, inducing helper T (Th2) cell development, promoting T cell proliferation and ultimately regulating T cell‐mediated immunity.^27^, ^28^

In the current review, we mainly focus on the hepatoprotective and immunoregulative effects of TCM and its natural products on several kinds of AIH‐related animal models. This review also provides a comprehensive summary of recent studies, highlights findings that have been ignored, and indicates potential prospects of clinical application of TCM for the management of AIH and associated manifestations.

## 2 MURINE INJURY MODELS FOR AIH

**FIGURE 1:**
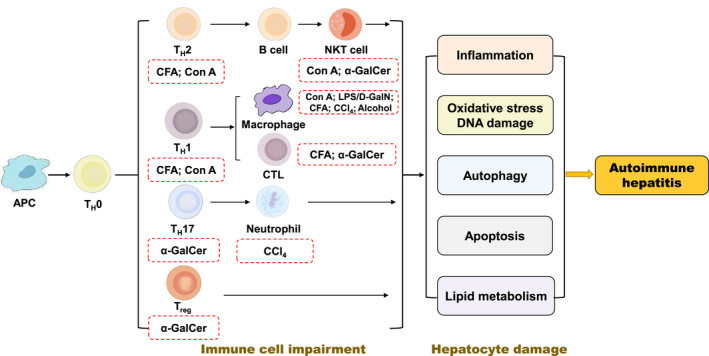
Different mechanisms account for the impairment of immune cells in autoimmune hepatitis (AIH)

Given that AIH pathogenesis is not yet been understood, multifarious animal models have been generated to mimic the histopathological features of AIH patients and help to identify mechanisms responsible for immunological intolerance. A variety of drugs has been associated with the induction of AIH, including concanavalin A (Con A), lipopolysaccharide/D‐galactosamine (LPS/D‐GalN), complete Freund's adjuvant (CFA), α‐galactosylceramide (α‐GalCer), carbon tetrachloride (CCl_4_) and alcohol (Figure 1). However, owing to differences in the major histocompatibility complex (MHC) class I and II proteins involved in the T cell response between mouse and human, mouse models used to mimic human autoimmune diseases often don't share the same target autoantigens and have certain limitations.^29^ Recently, Chinese herbal prescriptions, single Chinese herbs and active ingredients isolated from Chinese medicines have been shown to possess protective effects in these animal models of AIH and related diseases (Table 1).

**TABLE 1:** Effects of Chinese medicines on the treatment for autoimmune hepatitis (AIH) diseases

Animal models	TCM	Category	Effector immune cells	Target/signal pathways	References
Con A	PoPx	*Punica granatum*	NKT/CD4+/CD^8+^ T cells, neutrophils, macrophage	ALT, AST, TNF‐α, IFN‐γ and IL‐6	30
	Abietoquinones A	*Prunella vulgarisis*	CD3^+^ T cells, neutrophils, macrophage, splenocytes	IFN‐γ, TNF‐α, IL‐2, IL‐4, IL‐6, IL‐10, IL‐17	31
	Betulin	*Hedyotis hedyotidea*	NKT/CD4^+^/CD8^+^ T cells, neutrophils, macrophages and, splenocyte	CD69, TNF‐α, IFN‐γ, IL‐6	32
	PSA	*Periploca sepium* Bge	NKT/CD4^+^/CD8^+^ T cells, neutrophils, macrophage	ALT, IL‐4 and IFN‐γ	33
	YST	Decoction of herbal medicine	NKT/CD4^+^/CD8^+^ T cells, neutrophils, macrophage	a‐SMA, cola1(I), IL‐6, IL‐17, TGF‐β1, caspase‐3/8/9, Bax	34
	L‐THP	*Corydalis yanhusuo*	NKT/CD4^+^/CD8^+^ T cells, neutrophils, macrophages	TNF‐α, IL‐6, Bcl‐2, Bax, caspase 3/9, Beclin‐1, LC3‐II, TNF/TNFR, TRAF6/JNK signaling	35
	SCG	Decoction of herbal medicine	NKT/CD4^+^/CD8^+^ T cells, neutrophils, macrophage	ALT, AST, IL‐2/4/5, cytochrome P450, Bax, FAS, TRAIL, FASL, Caspase‐3/8/9	36, 37
	Resveratrol	*P. cuspidatum*	NKT/CD4^+^/CD8^+^ T cells, neutrophils, macrophage	TNF‐α, IFN‐γ, IL‐4, IL‐6, MCP‐1, SIRT1, Ki67, PCNA, Cyclin D1, Cdk2, p66^shc^	38
	RA	*Dracocephalum heterophyllum*	NKT/CD4^+^/CD8^+^ T cells, neutrophils, macrophages, leukocytes	ALT, AST, IFN‐γ, IL‐2, IL‐1β,IL‐10, AMPK, ACC	39
	Berberine	*Coptis chinensis*	NKT/CD4^+^/CD8^+^ T cells, neutrophils, macrophage	TNF‐α, IFN‐γ, IL‐6, IL‐10, STAT1, AMPK	40
	ERA	*Rabdosia amethystoides*	NKT/CD4^+^/CD8^+^ T cells, neutrophils, macrophage	IκB, p65, NF‐κB, TLR4	41
	ARS	Derivative	NKT/CD4^+^/CD8^+^ T cells, neutrophils, macrophage	IFN‐γ, IL‐17, IL‐1β, TNF‐α, ERK, JNK, p38, NF‐κB p65, IκBα	42
	ARC	*Arctium lappa* L.	NKT/CD4^+^/CD8^+^ T cells, neutrophils, macrophage	IL‐6, IL‐10, IFN‐γ, Atg7, Beclin1, LC3‐II, P62, Pkr, STAT1, Bnip3	43
LPS/D‐GalN	Scoparone	*Artemisia capillaris*	NKT/CD4^+^/CD8^+^ T cells, neutrophils, macrophage	TNF‐α, IL‐6, IFN‐β, TRIF, MyD88, IRF3, ERK, p38, JNK, I‐κB, TLR4	44
	Lupeol	*Adenophora triphylla* var. *japonica*	Monocytes, macrophages, hepatic Kupffer cells	TNF‐α, IL‐6, IRAK‐M, TRIF, TLR4 signaling	45
	Acacetin	*Agastache rugosa*	Monocytes, macrophages, hepatic Kupffer cells	TNF‐α, IL‐6, Atg5, Atg7, LC3‐II, p62, autophagic flux, TLR4 signaling pathways	46
	CTN	*Salvia miltiorrhiza* Bunge	Monocytes, macrophages, hepatic Kupffer cells	TAK1, JNK, ERK, p38,NF‐κb, caspase 3/8/9, cyto C	47
	HRP	Sea buckthorn (*Hippophae rhamnoides* L.)	Monocytes, macrophages, hepatic Kupffer cells	ALT, AST, MDA, SOD, GSH‐PX, TNF‐α, IL‐1β, MAPKs, NF‐κB/TLR4 signaling	48
	MA	Dry olive‐pomace oil	Monocytes, macrophages, hepatic Kupffer cells	MPO, TNF‐α, IL‐6, NF‐κB, Nrf2, HO‐1	49
	Alpinetin	Alpinia katsumadai Hayata	Monocytes, macrophages, hepatic Kupffer cells	MPO, MDA, TNF‐α, IL‐1β, NF‐κB, Nrf2, HO‐1	50
	Esculin	*Aesculus hippocastanum* L.	Monocytes, macrophages, hepatic Kupffer cells	MPO, MDA, TNF‐α, IL‐1β, NF‐κB, Nrf2, HO‐1	51
	CSS	*Acanthopanax sessiliflorus*	Monocytes, macrophages, hepatic Kupffer cells	ALT, AST, CAT, GSH, SOD, MDA, NF‐κB, Nrf2, HO‐1	52
	ADH	*Andrographis paniculata*	Monocytes, macrophages, hepatic Kupffer cells	ALT, AST, MDA, MPO, ROS, TNF‐α, IL‐1β, NF‐κB, Nrf2, HO‐1	53
	Isovitexin	*Oryza sativa*	Monocytes, macrophages, hepatic Kupffer cells	TNF‐α, ALT, AST, MPO, MDA, NF‐κB, Nrf2 signaling pathway	54
	SPG	*Panax ginseng*	Monocytes, macrophages, hepatic Kupffer cells	ALT, AST, MDA, SOD, CAT, GSH, TNF‐α, IL‐1β, P65, IκB, HO‐1, SIRT1/Nrf2/NF‐κB	55
	Biochanin A	Red clover and peanuts	Monocytes, macrophages, hepatic Kupffer cells	ALT, AST, IL‐1β, TNF‐α, MDA, Nrf2, HO‐1, NLRP3, TXNIP, ASC, caspase‐1	56
	TEN	*Polygala tenuifolia* root	Monocytes, macrophages, hepatic Kupffer cells	TNF‐α, IL‐1β, ALT, AST, MDA, MPO, ASK1, NF‐κB, MAPKs, Nrf2, HO‐1	57
	Linarin	*Chrysanthemum indicum* L.	Monocytes, macrophages, hepatic Kupffer cells	TNF‐α, IL‐1, IL‐6, FADD, caspase3/8, cyto C, STAT3, Bcl‐xL	58
	Garcinol	*Garcinia indica*	Monocytes, macrophages, hepatic Kupffer cells	MDA, NFκ‐B, Bax, caspase 3/8/9	59
CFA	PKRE	*Picrorhiza Kurroar hizome*	CD4^+^/CD8^+^ T cells, macrophage	TNF‐α, IL‐1β, IL‐6, IL‐10, COX‐2, TNFR1, iNOS, NF‐κB	60
	PRAP	*Paeoniae radix alba* polysaccharides	CD4^+^/CD8^+^ T cells, macrophage	NF‐κB, IL‐2, IL‐6 and IL‐10	61
	*Prunella vulgarisis*	*Prunella vulgarisis*	CD4^+^/CD8^+^ T cells, macrophage	IFN‐γ, IL‐17A, TGF‐β, BAX and caspase 3	62
α‐GalCer	Bu Xu Hua Yu recipe	Decoction of herbal medicine	NKT/Treg/Th17 cells	Foxp3, RORγt, IL‐10, IL‐17, TGF‐β	63
CCl_4_	LPCE	*Launaea procumbens* methanolice	Neutrophils	SOD, GST, GSR, GSH‐Px, GSH	64
	NPME	*Nicotiana plumbignifolia*	Neutrophils	TBARS	65
	TFs	*Cichorium intybus* L.	Macrophages, neutrophils	ALT, ALP, AST, GGT	66
	Ginsenoside Rg2	*Ginseng*	Neutrophils	AST, ALT, ALP, GSH, MDA	67
	Ucche	*Momordica charantia* L. var. muricata Willd.	Neutrophils, Kupffer cells	ROS, LPO, AST, ALT, TBARS, GPx, iron‐mediated signaling pathways	68
	TFs	*Cichorium glandulosum* seeds	Neutrophils	LPO, AST, ALT, ALP, TB, LDH, MDA, GSH, TBARs	69
	SP	*Hippophaerhamnoides L*.	Macrophages, neutrophils	ALT, AST, SOD, MAD, GSH, GSHpx, TNF‐α, IL‐1β, NO, iNOS, TLR4, NF‐κB, MAPKs	70
	Apocynin	*Picrorhiza kurroa*	Neutrophils	ALT, AST, ROS, MPO, MDA, SOD, NO, APOP	71
	SME	*Sonchus arvensis*	Neutrophils	CAT, SOD, GST, GSR, GSHpx, TBARS, DNA	72
Alcohol	PG	*platycodon grandiflorum*	Lymphocytes, macrophages	CAT, SOD, GPx, GR, GOT, GPT, TG, ROS.	73
	Cii	*Cichorium intybus*	Lymphocytes, macrophages	GOT, GPT, TG, CYP2E1, ADH, ALDH	74
	PCP	*Penthorum chinense* Pursh	Lymphocytes, macrophages	TNF‐α, IL‐6, MDA, SOD, GSH, ROS, GPx, HO‐1, CYP2E1, TG, Nrf2	75
	Taraxasterol	*Taraxacum*	Lymphocytes, macrophages	ALT, AST, MDA, GSH, SOD, TG, TNF‐α, IL‐6, CYP2E1/Nrf2/HO‐1, NF‐κB signaling pathways	76
	PCP	*Cichorium intybus*	Lymphocytes, macrophages	ALT, AST, MDA, GSH, SOD, CAT, WAT, CYP2E1, CD36, ATGL	77
	Gastrodin	*Gastrodia elata* Bl.	Lymphocytes, macrophages	ALT, AST, MDA, SOD, GPx, CAT, CYP2E1, TNF‐α, IFN‐γ, IL6, CXCL‐1, VCAM‐1, NF‐κB, AMPK/Nrf2 pathway	78
	TASP	*Triticum aestivum*	Lymphocytes, macrophages	ALT, AST, GSH, SOD, CYP2E1, HO‐1, Bcl‐2/Bax, PI3K/Akt/Nrf2 signaling pathway	79
	AC	*Cinnamomea* mycelia	Lymphocytes, macrophages	Akt/NF‐κB signaling pathway, caspase 3/8/9	80
	ME	*Morchella esculenta*	Lymphocytes, macrophages	YKL‐40, IL‐7, PAI‐1, RBP4, Keap‐1, Nrf2, NF‐κB signaling	81

### 2.1 Effects of TCM on the treatment of immune cell‐mediated murine hepatic models

#### 2.1.1 TCM and Con A‐induced liver injury models

Con A, a lectin isolated from jack beans, is a T cell mitogen for inducing antigen non‐specific T cell activation and severe acute hepatitis with cytokine storm.^82^ Recent studies report that Con A can stimulate CD4^+^ T cells, natural killer T (NKT) cells, macrophages and APCs, and cause the secretion of proinflammatory cytokines including interleukins (IL‐6, IL‐12, IL‐18), tumor necrosis factor‐α (TNF‐α), interferon‐γ (IFN‐γ) and several chemokines in mice.^83^ Although Con A represents a model of non‐specific and T cell‐mediated acute liver injury rather than a typical AIH model, Con A‐induced hepatitis has been extensively used to investigate the immune system‐mediated liver injury and evaluate possible therapies for human AIH.

According to their different biological functions in the immune response, T cells are further divided into three subgroups: cytotoxic T cells (CTLs), namely CD8^+^ T cells, which release granzyme and perforin to induce the apoptosis of target cells; CD4^+^ helper T cells (Th cells), which secrete a variety of cytokines such as interleukins, TNF‐α and IFN‐γ and activate B cells to differentiate and produce antibodies; regulatory T cells (Tregs), which inhibit the immune response by secreting forkhead box p3 (Foxp3), transforming growth factor‐β (TGF‐β), IL‐4, and IL‐10.^84^, ^85^, ^86^ It has been well‐established that aberrant activation of CTLs, cytokines released by Th1 cells and macrophages, and adhesion of NKT cells to hepatocytes coated with autoantibodies contribute to hepatocyte destruction in Con A‐induced animal models.^87^ Wang et al.^30^ obtained pomegranate peel extract (PoPx) from *Punica granatumand* and demonstrated that pretreatment in a Con A‐induced AIH murine model with PoPx obviously reduced the level of cytokines (TNF‐α, IFN‐γ and IL‐6), serum transaminases and the murine mortality rate, and played a protective role by decreasing the liver damage caused by ROS. It also remarkably reduced the hepatic infiltration of activated CD4^+^/CD8^+^ T cells. Yunfang Cao isolated abietoquinones A from *Prunella vulgarisis* and found that it decreased the serum level of aminotransferases, inhibited the production of proinflammatory cytokines and alleviated the proliferation and infiltration of CD3^+^ T cells in a Con A‐induced hepatitis mouse model.^31^ NKT cells, a T cell subset expressing surface markers of both NKT cells and T lymphocytes, are crucial first responders against multiple infectious diseases. Once stimulated, NKT cells secrete a large number of cytokines and chemokines, especially IL‐4 and IFN‐γ, and simultaneously inhibit the differentiation of Th1/Th2 cells.^88^ It has also been reported that betulin, extracted from *Hedyotis hedyotidea*, inhibited the activation of NKT and conventional T cells by decreasing the enhanced expression of CD69, prevented the production of pro‐inflammatory cytokines, particularly Th1‐type cytokines, in these two cell populations, and subsequently ameliorated liver injury.^32^ Additionally, betulin abolished NKT cell death in mice, and dose‐dependently attenuated splenocyte proliferation stimulated by Con A in vitro.^32^ Researchers from the same lab further extracted periplocoside A (PSA) from *Periploca sepium* Bge and investigated whether this ingredient also alleviated Con A‐induced hepatitis in mice. As expected, PSA inhibited the proliferation of primary T cells and the production of NKT‐derived inflammatory cytokines (IL‐4 and IFN‐γ), reduced the necrosis and infiltration of leukocytes and protected mice from Con A‐induced AIH damage.^33^

Apoptosis is programmed cell death including internal and external apoptosis pathways and is of great importance in resisting pathogen invasion and regulating the immune response. In addition to its role in stimulating T cells, Con A can cause AIH injury by directly triggering hepatic parenchymal cell death. The intrinsic pathway includes endoplasmic reticulum (ER) stress or mitochondrial dysfunction. After the destruction of Ca^2+^ balance or the aberrant accumulation of ER protein, endoplasmic reticulum stress (ERS) triggers the apoptosis signals and promotes cell apoptosis by activating caspase 3/7/12.^89^ Under the stimulation of various apoptotic signals, B‐cell lymphoma‐2 (Bcl‐2) and Bcl‐2‐associated X protein (Bax) form a heteromer and migrate to the outer membrane of mitochondria, causing the release of cyto C and a cascade reaction of downstream caspases.^90^ Yongdamsagan‐tang (YST) extracts markedly inhibited the apoptosis of hepatocytes and prevented the Con A‐induced AIH injury in mice via the suppression of caspase‐3/8/9 and Bax.^34^ In addition, YST also suppressed IL‐6 expression and lipid accumulation caused by IL‐17 in hepatocytes. Levo‐tetrahydropalmatine (L‐THP), an active ingredient from *Corydalis yanhusuo*, was reported to inhibit T lymphocyte proliferation and cytokine production, upregulate Bcl‐2 expression, downregulate Bax and caspase 3/9 through the inhibition of TNF‐α‐mediated TNF receptor associated factor 6/c‐Jun N‐terminal kinase (TRAF6/JNK) signaling pathway, thereby reducing Con A‐induced hepatocyte apoptosis.^35^ Sancao granule (SCG), a traditional Chinese formula, has long been used for treating AIH in the clinic. There is renewed interest in SCG‐induced protective effects, which decrease pro‐inflammatory cytokines and inhibit hepatocyte death. Using metabolomics profiling, SCG was found to ameliorate Con A‐induced liver injury in mice by inhibiting cytoplasmic vacuolization, hepatocyte apoptosis and neutrophil infiltration in livers, and the metabolites related to the efficacy of SCG in treating AIH were clarified.^36^ An increasing number of studies have recently implied that external factors promote the combination of death ligand and its receptor to form an apoptosis‐inducing complex on the cell membrane, which causes the cascade reaction leading to apoptosis. Other research has shown that oral administration of SCG in Con A‐induced mouse model regulated the balance of pro‐ and anti‐inflammatory cytokines and dose‐dependently inhibited the activation of apoptotic mediators (Bax, Caspase‐3/8/9) by interrupting the tumor necrosis factor‐related apoptosis inducing ligand (TRAIL)‐ and first apoptosis signal ligand (FASL)‐mediated apoptosis pathway, which subsequently decreased the pathological changes in liver tissue.^37^ Furthermore, SCG was reported to restrain neutrophil infiltration of liver tissues by adjusting the level of cytokines (IFN‐γ, TNF‐α, IL‐2, IL‐4, IL‐5 and IL‐33), decreasing the high expression of cytochrome P450, and increasing the low expression of FAS in a Con A‐induced liver injury of mice.^37^

Oxidative stress was reported to result in the dysregulation of the immune system, abnormal activation of cell‐death signals and the production of autoantibodies.^91^ P66^shc^ acts as a dual player in cell survival and the interplay between p66^shc^ and oxidative stress is implicated in the pathogenesis of various liver diseases, including AIH.^92^ Silent information regulator 1 (SIRT1) is an NAD^+^‐dependent histone deacetylase and regulates cell apoptosis, inflammation and oxidative stress, which also influences the function of multiple biological targets such as p66^shc^. Resveratrol, a well‐known polyphenol phytoalexin, has been isolated in the roots of multitudinous Chinese herbal medicines, such as *Polygonum cuspidatum*, *Magnolia officinalis* Rehd. and *Morus alba* L. and acts as a hepatoprotective agent against multiple xenobiotics‐induced liver injury. Recent studies have shown that resveratrol functions as an activator of S1RT1.^93^ Recently, resveratrol was reported to dramatically reduce the infiltration of macrophages, neutrophils, and T lymphocytes in the liver caused by Con A. Furthermore, it inhibited Con A‐induced reactive oxygen species (ROS) production and hepatocyte apoptosis in mice by decreasing the level of p66^shc^ and increasing the expression of SIRT1.^38^ SIRT1 also exerts its anti‐inflammatory, immunosuppressive and other biological activities by regulating a series of survival‐related signaling pathways, such as adenosine 5′‐monophosphate (AMP) activated protein kinase (AMPK). It has been demonstrated that rosmarinic acid (RA) reduced hepatocyte swelling and death, infiltration of leukocytes and serum levels of IFN‐γ, IL‐2 and IL‐1β, and elevated the serum level of IL‐10 by inducing AMPK activation and subsequent acetyl‐CoA carboxylase (ACC) phosphorylation, thereby protecting mice against Con A‐induced AIH.^39^ In addition, berberine decreased the production of TNF‐α, IFN‐γ, IL‐2, IL‐1β, increased the level of IL‐10 and alleviated hepatitis by blocking the transcriptional activity of signal transducer and activator of transcription 1 (STAT1), which was largely dependent on activating AMPK.^40^

The transcription factor nuclear factor‐kappa B (NF‐κB) is considered a central mediator of the immune response and exists as an inhibitor of nuclear factor kappa B (IκB) kinase (IKK) complex with its inhibitory protein α (IκBα) in the cytoplasm in a quiescent condition. Once activated, NF‐κB is translocated from the cytoplasm to the nucleus, driving the expression of target genes involved in immune and inflammation responses.^94^ It was reported that SIRT1 negatively regulated the transcriptional activity of NF‐κB/P65 and thus controlled the immune response of macrophages.^38^ Interestingly, the higher level of NF‐κB/P65, lower expression of SIRT1, accumulated macrophages and hepatocyte apoptosis that appeared in the livers of aged mice following long‐term administration of Con A were all completely reversed by resveratrol pretreatment.^95^ An extract of *Rabdosia amethystoides* (Benth) Hara (ERA) was reported to significantly inhibit the release of pro‐inflammatory cytokines, attenuate the activation of the NF‐κB pathway by reducing the expression of Toll‐like receptors (TLRs) and inhibit the phosphorylation of IκB and P65 in Con A‐induced mouse liver injury.^41^ In addition, mitogen‐activated protein kinases (MAPKs) pathways including the extracellular signal‐related proteins kinase (ERK), JNK and mitogen‐activated protein kinase38 (p38) play a striking role in the regulation of pro‐inflammatory cytokine production and the immune system.^96^ It was reported that artesunate (ARS), a derivative of artemisinin, possesses a higher solubility, shorter half‐life and better anti‐inflammatory activity than artemisinin itself. Lijingui et al.^42^ reported that ARS reduced the excess production of pro‐inflammatory cytokines (IFN‐γ, IL‐17 and TNF‐α), increased the production of IL‐10, suppressed the level of serum aminotransferases and protected mice from Con A‐induced autoimmune hepatitis by inhibiting the phosphorylation of ERK, JNK and p38, NF‐κB p65 and IκBα signaling in a dose‐dependent manner.

Autophagy is a cannibalistic catabolic process involving the degradation of cytoplasmic constituents in lysosomes, and contributes to most biological processes from cell apoptosis and aging to immune reactions.^97^ Conserved factors called autophagy‐related gene (ATG) proteins and light chain 3 (LC3), as well as Beclin‐1 and autophagy substrate p62 are required for autophagosome formation. IFN‐γ was demonstrated to upregulate the expression of many autophagy‐related proteins such as double‐stranded RNA‐dependent protein kinase (Pkr), Atg7, LC3‐II, p62 through the Janus Kinase 1(JAK1)/STAT1 signaling pathway in Con A‐induced hepatitis.^98^ After induction by IL‐6/JAK/STAT3 signaling, bnip3, a pro‐apoptotic protein, activated Bcl‐2‐antagonist/killer (BAK)/BAX and initiated mitochondrial apoptosis in Con A‐induced hepatitis.^99^ Arctigenin (ARC), a biologically active lignan extracted from the seeds of *Arctium lappa* L, inhibited autophagy by downregulating Atg7, Beclin‐1, LC3‐II and p62, and prevented hepatocyte apoptosis by decreasing the expression of Bnip3 and Beclin‐1 in Con A‐induced AIH injury. In addition, ARC significantly inhibited the release of cytokines and portal inflammation, via a possible molecular mechanism inhibiting IFN‐γ/IL‐6/Stat1 and IL6/Bnip3 signaling pathways.^43^

#### 2.1.2 TCM and LPS/D‐GalN‐induced liver injury models

Recently, a variety of studies have shown a strong correlation between endotoxin LPS and liver failure. LPS stimulates the excessive secretion of inflammatory cytokines through the activation of TLR4 and NF‐κB signaling, thereby leading to systemic inflammation and multiple organ failure.^100^ D‐GalN, a hepatocyte‐specific macromolecule synthesis inhibitor, significantly aggravated LPS‐induced liver inflammation in mice. LPS/D‐GalN‐induced hepatotoxicity is related to the upregulation of pro‐inflammatory cytokines and the generation of free radicals that readily elicit a distinct DNA damage response and cause significant cytotoxicity. Although acute co‐injection of LPS/D‐GalN is a widely used experimental method for mimicking acute liver injury, there are still several studies that apply this animal model to investigate the therapeutic effects and molecular mechanism of herbal medicines on AIH.

TLR4 activates MAPK and NF‐KB signaling by interacting with the connector protein myeloid differentiation factor (MyD88), or induces the phosphorylation of interferon regulatory factor 3 (IRF3) and the expression of IFN‐β by activating TIR‐domain‐containing adapter‐inducing IFN‐β (TRIF) and TRAF3. LPS/D‐GalN was observed to induce the activation of MyD88 and TRIF‐dependent signaling pathways, which further increased serum ALT activity and inflammatory cell infiltration and caused fulminant hepatic failure in mice. However, most of the above symptoms caused by LPS/D‐GalN were improved after scoparone treatment, a major component of *A*. *Capillaris*, accompanied by inhibition of the expression of IFN‐β, TNF‐α, IL‐6, TRIF, MyD88 and the phosphorylation of IRF3, ERK, p38, JNK and I‐κB.^44^ Interleukin‐1 receptor‐associated kinase M (IRAK‐M) is a member of the IRAK family that acts as a negative regulator of TLR signal transduction. Researchers have suggested that lupeol, a major active triterpenoid isolated from *Adenophora triphylla* var. *japonica*, plays an important role in restraining TLR4/MyD88 inflammatory signaling through the augmentation of increased IRAK‐M, thus further alleviating LPS/D‐GalN‐mediated liver injury.^45^ A previous study demonstrated that acacetin, a flavonoid isolated from *Agastache rugosa*, not only inhibited TLR4‐mediated inflammatory responses and hepatocellular necrosis, but also enhanced autophagic flux to protect against LPS/D‐GalN‐induced liver injury^46^. TAK1, together with its coactivator TAB1, facilitates the expression of inflammatory cytokines by simultaneously triggering the NF‐κB and MAPK pathways.^101^ Cryptotanshinone (CTN) inhibited LPS/D‐GalN‐induced activation of NF‐κB and the release of inflammatory cytokines (IL‐1α and IL‐β) by suppressing the phosphorylation of TAK1 and MAPK pathways, and attenuated apoptosis by blocking the activation of caspase 3/8/9 as well as the release of cyto C from the mitochondria.^47^

Emerging evidence increasingly suggests the critical role of dysregulation of oxidative stress played in LPS/D‐GalN‐induced AIH. Pretreatment of the polysaccharide extracts of Sea buckthorn (HRP) significantly inhibited the activation of NF‐κB, reduced the numbers of p65 positive cells and improved abundant inflammatory cell infiltration in livers through the TLR4‐MyD88 signaling pathway, and restored the activity of antioxidant enzymes and prevented the necrosis of hepatocytes, thereby inhibiting LPS/D‐GalN‐induced liver injury in mice.^48^ Nuclear factor‐E2 related factor 2 (Nrf2) is generally induced by accumulated ROS in oxidative stress, followed by translocation to the nucleus and binding to antioxidant response elements (ARE) to promote the transcription of antioxidant genes including heme oxygenase‐1 (HO‐1) and inhibit NF‐κB activity in hepatocytes. A variety of drugs including alpinetin/esculin/andrographolide/maslinic acid/isovitexin/chiisanoside were reported to alleviate LPS/D‐GalN‐induced histopathological changes and reduce liver inflammation by inhibiting NF‐κB and activating Nrf2 signaling pathway.^49^, ^50^, ^51^, ^52^, ^53^, ^54^, ^102^ Another study further suggested that SIRT1 was associated with the regulation of the inflammatory response and oxidative stress by deacetylating Nrf2 and NF‐κB.^103^ Sesquiterpenoids from *Panax ginseng* (SPG) dose‐dependently attenuated neutrophil enrichment, inhibited the phosphorylation of P65 and IκB, as well as levels of TNF‐α and IL‐1β, which further promoted the nuclear translocation of Nrf2 and expression of HO‐1 by activating the SIRT1 signaling pathway in the LPS/GalN‐induced AIH mouse model.^55^ Furthermore, Nrf2 has been reported to inhibit the activation of NOD‐like receptor protein 3 (NLRP3) inflammasome, caspase‐1 cleavage and IL‐1β production,^104^ which are activated under the stimulation of LPS/D‐GalN. Recent reports suggest that the inhibition of NLRP3 inflammasome suppressed liver hepatitis in the LPS/D‐GalN‐induced mouse model. Notably, biochanin A was found to inhibit LPS/GalN‐induced extensive hemorrhage, inflammatory cytokines and neutrophil infiltration by dose‐dependently up‐regulating the expression of Nrf2 and HO‐1 in mice. Although it didn't directly inhibit the expression of NLRP3, biochanin A markedly inhibited the upregulation of thioredoxin interacting protein (TXNIP), the interaction of NLRP3 with apoptosis associated speck‐like protein (ASC) and caspase‐1, as well as the interaction between TXNIP and NLRP3.^105^

Apoptotic signal‐regulated kinase 1 (ASK1) is activated in response to various stresses and regulates inflammation and apoptosis by activating MAPK pathways. Tenuigenin (TEN), a major active component of *polygala tenuifolia* root, protected against LPS/GalN‐induced extensive hemorrhage, necrosis and neutrophil infiltration by upregulating Nrf2 and HO‐1 and inhibiting ASK1, NF‐κB and MAPKs signaling.^57^ On the one hand, LPS/GalN promoted the binding of apoptotic signaling sensor (TNF‐α/TNFR1 complex) to FADD, which triggers caspase 8 and leads to the cascade reaction of caspase, such as caspase 3.^106^ Seok‐joo Kim reported that linarin, isolated from *Chrysanthemum indicum* L., inhibited the expression of Fas‐related death domains and caspase 8, further reduced the release of cytochrome, and alleviated extensive areas of necrosis and portal inflammation caused by LPS/GalN.^58^ Co‐administration of LPS and GalN was also reported to increase the cytosolic release of cyto C as well as the activity of caspase 3/8/9. Garcinol, a polyisoprenylated benzophenone derivative of *Garcinia indica*, significantly reduced the level of the acetylated p65 subunit of NF‐κB, restrained the cleavage of caspase 3, and decreased the activity of caspase 3/9 and the expression of Bax and Bax/Bcl‐2 ratio in liver tissue, thereby resulting in the inhibition of LPS/D‐Gal induced hepatocyte apoptosis and the inflammatory response.^59^

#### 2.1.3 TCM and CFA‐induced liver injury models

Stimulation of the immune system with CFA results in altered leukocyte proliferation and differentiation, antibody production and inflammatory responses through the continuous release of immunogen. The use of CFA is necessary for the induction of several experimental mouse models of autoimmune diseases, including but not limited to autoimmune neuritis, uveitis and encephalomyelitis.^107^ CFA has also been applied to establish mouse models mimicking the pathological features of patients with AIH, owing to its capacity to elicit immune responses. However, CFA‐induced multiple organ damage further limits its application in experimental research. Therefore, most recent studies tend to combine recombinant CYP2D6, a hepatic protein target of anti‐LKM1 antibodies, with CFA to induce an experimental AIH model.

It is reported that CFA‐immune responses depend on the function of various cells such as macrophages, which phagocytose and digest cell fragments and pathogens and regulate the release of TNF‐α, IL‐1β, IL‐6, and cyclooxygenase‐2 (COX‐2) to activate other immune cells and maintain immune homeostasis. Recently, *Picrorhiza Kurroar* hizome extract (PKRE) was noted to inhibit the production of proinflammatory factors TNF‐α, IL‐1β, IL‐6, TNFR1 and COX‐2 in CFA‐induced peritoneal macrophages. Moreover, it markedly inhibited nitric oxide synthase (iNOS) and the phosphorylation of NF‐κB by blocking the activation of IκB in activated macrophages.^60^ IL‐2 and IL‐6/IL‐10 are produced by CD4^+^ Th1 cells and CD4^+^ Th2 cells, respectively, expression of which reflects the severity of immune inflammatory reaction‐caused hepatocyte damage. Wang et al. found that *Paeoniae radix alba* polysaccharides (PRAP) significantly alleviated cellular fatty degeneration, and improved cellular hydropic degeneration and hepatocyte regeneration. It also reduced the infiltration of CD4/8^+^ T cells and inhibited the upregulation of inflammatory cytokines IL‐2 and IL‐6, and the downregulation of IL‐10 in a CFA‐treated mouse model, which was dependent on the downregulation of NF‐κB signaling pathway.^61^ Similar to other AIH experimental models, the pathogenesis of CFA‐induced immune hepatitis is associated with hepatic parenchymal cell apoptosis. Recently, *Prunella vulgarisis* was reported to improve the CFA‐induced hepatic inflammation and hepatocyte necrosis by reducing the infiltration of inflammatory cells, decreasing the level of pro‐inflammatory cytokines IFN‐γ and IL‐17A and increasing the production of anti‐inflammatory cells, Treg cells, and cytokines, TGF‐β. On the other hand, it protected hepatocytes from CFA‐caused apoptosis by directly inhibiting BAX and caspase 3.^62^

#### 2.1.4 TCM and α‐GalCer‐induced liver injury models

α‐GalCer is a glycolipid isolated from a marine sponge, which specifically activates iNKT cells, secretes cytokines such as IFN‐γ, TNF‐α, IL‐6, and IL‐10, and thus results in AIH injury in vivo. The α‐GalCer‐induced AIH mouse model is associated with increased levels of pro‐inflammatory cytokines produced by NKT cells, the infiltration of inflammatory cells and the necrosis of local hepatocytes. Furthermore, pretreatment with α‐GalCer aggravated D‐GalN‐mediated AIH in mice. After induction by IL‐6 and retinoic acid receptor‐related orphan receptor gamma‐t (RORγt), Th17 cells produce IL‐17 and promote it attached to its receptor and further trigger inflammation through the MAPK and NF‐κB pathway. On the other hand, IL‐10 and Foxp3 induce the proliferation of Tregs, which modulates the immune system, maintains tolerance to self‐antigens and thus alleviates AIH damage caused by secretion of TGF‐β. The inhibition of IL‐17 enhances the immunosuppressive activity of Treg cells, thus promoting its differentiation. Wang et al. proved that the Bu Xu Hua Yu recipe positively up‐regulated the secretion of IL‐10 and Foxp3, downregulated the secretion of IL‐17, regulated the development and proliferation of Treg/Th17 cells, and subsequently improved α‐GalCer‐induced hepatitis in mice.^63^

### 2.2 Effects of TCM on the treatment of hepatotoxicity‐mediated murine hepatic models

#### 2.2.1 TCM and CCl_4_‐induced liver injury models

CCl_4_ is a well‐known hepatotoxin and induces either acute or chronic hepatitis. Oral administration of CCl_4_ results in the activation of leukocyte and immune‐mediated liver damage, and is thus regarded as an experimental model of AIH. Although CCl_4_ efficiently stimulates the production of pro‐inflammatory cytokines, disrupts liver tolerance and even triggers autoimmune responses in livers, a few studies have pointed out that CCl_4_ may induce a non‐specific liver inflammation rather than a typical AIH.^56^ Previous studies reported the hepatoprotective role in the CCl_4_‐induced hepatitis mouse model of total flavonoids (TFs) from *Cichorium glandulosum* seeds, chloroform extract of *Launaea procumbens* (LPCE), *Nicotiana plumbignifolia* methanolice extract (NPME), and ginsenoside Rg2.^64^, ^65^, ^66^, ^67^ With prolonged CCl_4_ treatment, lipid peroxidation (LPO) in response to excessive accumulation of free radicals results in severe damage to the cell membrane and eventually necrosis. Due to a deficiency of heme oxygenase, Kupffer cells and hepatocytes are not capable of clearing iron deposition, which then results in oxidative damage and hepatitis by the Fenton reaction and aggravates LPO. Ucche (*Momordica charantia* L. var. muricata (Willd.) Chakravarty) reduced the infiltration of inflammatory cells and LPO by inhibiting iron‐mediated signaling pathways and activating hematopoietic stem cells in a CCl_4_‐induced AIH rat model.^68^

Nitric oxide (NO), produced by iNOS, is another critical mediator of hepatic inflammation, acting by regulating macrophages and neutrophils.^108^ TFs markedly inhibited the levels of advanced protein oxidation products (APOP) and NO, as well as the activities of LPO and pancreatic lipase, which protected the liver from CCl_4_‐induced toxicity and inflammation.^69^ Similarly, seabuckthorn berry polysaccharide pretreatment also prevented CCl_4_‐induced hepatitis damage by decreasing the expression of NO, iNOS and TLR4, and inhibiting the production of cytokines by macrophages and the phosphorylation of NF‐κB and MAPK signaling.^70^ After recruitment and activation of phagocytes, myeloperoxidase (MPO), a neutrophil marker, is highly expressed in neutrophils, participating in the generation of ROS. Apocynin, from the plant *Picrorhiza kurroa*, was demonstrated to significantly restore the activity of SOD, reduce the level of malondialdehyde (MDA), MPO, NO, and APOP, and inhibit vacuolar degeneration and massive infiltration of neutrophils and other immune cells in a CCl_4_‐induced AIH mouse model.^71^

Emerging evidence also highlights the potential interaction between AIH and drug‐induced DNA damage. Innate immune receptors (TLRs and non‐TLRs) recognize the immunogenicity of oxidized DNA in ROS‐induced damage.^91^ The radical of CCl_4_ was reported to directly contribute to the fragmentation and oxidative damage of DNA and hepatic inflammation, which were significantly restored towards normal levels with the administration of *Sonchus arvensis* (SME).^72^

#### 2.2.2 TCM and alcohol‐induced liver injury models

Considering its increased clinical incidence, alcohol is regarded as another potential trigger of liver autoimmunity.^109^ Excessive alcohol and its metabolites such as acetaldehyde and malondialdehyde (MAA) give rise to the production of autoantibodies and cause inflammatory responses. Emerging evidence demonstrates that MAA synergistically forms an adduct with soluble proteins, which promotes the release of cytokines, the proliferation of T cells and the production of antibodies.^110^ However, the mechanisms by which MAA adduction disrupts immune tolerance and induce autoimmune responses remain unclear.

Cytochrome P450 (CYP) 2E1‐mediated alcohol metabolism leads to excessive production of ROS (·OH, H_2_O_2_, $O_{2}^{‐}$), oxidative stress, LPO and hepatocyte apoptosis.^111^ It has been reported that *platycodon grandiflorum* inhibited the activity of SOD and CAT, restrained the production of MDA, alleviated a high accumulation of microvesicular‐type fat in the hepatocyte cytoplasm and prevented alcohol‐induced oxidative stress and acute hepatitis.^73^ It was also reported that *Cichorium intybus* root extract reduced lipid accumulation and alleviated alcohol‐induced liver injury by upregulating alternative alcohol metabolism pathways mediated by ADH and ALDH and inhibiting the levels of GOT, GPT, TG and CYP2E1.^74^ Under the stimulation of alcohol, excessive accumulation of fatty acids resulted in activation of lymphocytes, infiltration of macrophages and hepatic inflammation.^112^ *Penthorum chinense* Pursh (PCP) reduced oxidative stress, improved inflammation and protected the liver from acute alcohol‐induced hepatitis by activating the Nrf2/HO‐1 pathway and downregulating the expression of CYP2E1 and hepatic TG levels.^75^ A previous study showed that taraxasterol effectively improved hepatocyte steatosis and prevented the development of fatty liver by significantly reducing TG content, and inhibiting the secretion of pro‐inflammatory cytokines (TNF‐α and IL‐6) via CYP2E1/Nrf2/HO‐1 and NF‐κB signaling pathways in alcohol‐induced liver injury in mice.^76^ Notably, pretreatment with PCP inhibited the upregulation of fatty acid translocation enzyme (CD36) expression, and ameliorated dysfunctional white adipose tissue derived‐fatty acid influx to the liver, thereby protecting the liver from acute alcohol‐induced hepatitis.^77^

Inflammatory infiltration and hepatocyte apoptosis are widely observed in the alcohol‐induced AIH mouse model. It is well established that alcohol and its metabolites promote the release of cytokines and experimental hepatitis, which are closely related to the activation of NF‐κB signaling. Gastrodin ameliorated histopathological lesions of hepatocytes, alleviated liver inflammation and reduced the level of cytokines and chemokines, such as TNF‐α, IFN‐γ and IL‐6, chemokine (C‐X‐C motif) ligand 1 (CXCL‐1), vascular cell adhesion molecule 1 (VCAM‐1) in a dose‐dependent manner by regulating the NF‐κB and AMPK/Nrf2 pathways in alcohol‐induced AIH.^78^ Akt acts as a critical upstream activator in the inflammatory reaction involving Nrf2 nuclear translocation, NF‐κB activation and ROS generation.^113^ *Triticum aestivum* sprout‐derived polysaccharide protected against alcohol‐induced hepatitis by inhibiting the phosphatidylinositol 3‐kinase (PI3K)/phosphor‐protein kinase B (Akt) signaling pathway and regulating Nrf2 nuclear translocation.^79^ Another study reported that *cinnamomea* mycelia (AC) strongly reduced lipid droplet formation, hepatocyte apoptosis and inflammatory infiltration in livers by suppressing the increased levels of TNF‐α, inhibiting the phosphorylation of Akt and NF‐κB and suppressing caspase 3/8/9 signaling in an alcohol‐exposed rat model.^80^ Under physiological conditions, excessive alcohol downregulates the production of retinol‐binding protein 4 (RBP4), upregulated the expression of inflammatory factors including chitinase‐3‐like protein 1 (YKL 40) and plasminogen activator inhibitor type 1 (PAI‐1) in the liver.^114^ A recent study further reported that *Morchella esculenta* prevented inflammatory cell infiltration, inhibited the production of YKL‐40, IL‐7, PAI‐1 and RBP4 and improved the alcohol‐induced imbalance in pro‐oxidative and anti‐oxidative signaling by altering Nrf2 and NF‐κB pathways in the liver.^81^, ^115^

## 3 CONCLUSION AND PROSPECT

AIH is a chronic inflammatory disorder characterized by interface hepatitis, increased serum autoantibodies and hypergammaglobulinemia, representing a major health burden without any available cure. Many animal models for human AIH have been generated to either provide an appropriate approach to investigate regulatory immune mechanisms or evaluate possible therapeutics. However, based on all the evidence summarized above, almost all these animal models of AIH have obvious limitations that raise the difficulty of exploring AIH pathogenesis. Although the Con A‐related hepatic injury is an extensively used model mimicking the histopathological changes of human AIH, it is more suitable for studying the underlying mechanisms of immune activation and/or regulation. Moreover, while chemicals such as CCl_4_ are capable of triggering liver inflammation and disrupting immune tolerance, they are more likely to induce a non‐specific immune response. In addition, there is controversy about whether a liver injury model induced by MAA with soluble proteins can be widely applied to reflect the AIH injury in humans, due to the loss of immunogenicity and lack of detailed mechanisms. Furthermore, the feasibility of several established AIH animal models is still questionable. Collectively, several phenotypic characteristics need to be fulfilled in generating any novel AIH models in the future: firstly, inducing the production of autoantibodies against liver antigens; secondly, regulating the serum levels of inflammatory factors such as cytokines and chemokines; finally, altering the levels of T‐cell subtypes or triggering the disorder of the immune system.

Emerging evidence shows that Chinese medicine prescriptions and natural products isolated from TCM provide additional benefits in the prevention of chronic liver diseases, including AIH, and represent a critical source of drug screening and development. In the current review, we comprehensively reviewed these researches. However, most of the above studies only investigated the regulatory effects of TCM on the typical inflammatory signaling pathways (especially those for NF‐κB/MAPKs) and inflammatory factors (like IFN‐γ/TNF‐α/IL‐6), without detecting the changes of lgG/autoantibodies and T cell subsets in the respective AIH animal models. Therefore, the precise mechanism of the protective effects of the above‐reported TCM components against AIH still requires extensive research. Considering the existing deficiencies in the current AIH animal models, researchers should test combinations of different drugs or construct new animal models. For example, the NTxPD‐1^−/−^ mouse model is an inducible AIH model that lacks the programmed cell death 1 (PD‐1) gene and a sufficient number of regulatory T cells to maintain the immune T cell balance.^116^ Another novel AIH model is the CYP2D6 mouse model, which is generated by the infection of an adenovirus encoding for human CYP2D6, the best‐characterized autoantigen recognized by T cells and antibodies of AIH‐2 patients.^117^ These novel animal models have not been summarized in this review, because, to date, no research has yet investigated the hepatoprotective effects of TCM on these models. Further comprehensive evaluations of the effects and mechanisms of TCM on AIH‐related animal models and identification of specific molecular targets are urgently needed.

## CONFLICT OF INTEREST

The authors have declared no conflict of interest.

## AUTHORS’ CONTRIBUTIONS

XL conceived the original idea and supervised the study. XL, JL, ZM and Han Li prepared the manuscript and figures. All authors have approved the final manuscript.
